# *In Vitro* Vitamin K_**3**_ Effect on Conjunctival Fibroblast Migration and Proliferation

**DOI:** 10.1155/2014/916713

**Published:** 2014-01-08

**Authors:** I. Pinilla, L. B. Izaguirre, F. J. Gonzalvo, E. Piazuelo, M. A. Garcia-Gonzalez, A. I. Sanchez-Cano, F. Sopeña

**Affiliations:** ^1^Department of Ophthalmology, Lozano Blesa University Hospital, C/San Juan Bosco 15, 50009 Zaragoza, Spain; ^2^Aragon Institute of Health Sciences (IIS Aragon), 50009 Zaragoza, Spain; ^3^Department of Ophthalmology, Hospital García Orcoyen, Navarra, 31200 Estella, Spain; ^4^Department of Ophthalmology, Complejo Hospitalario de Navarra, Navarra, 31008 Pamplona, Spain; ^5^Department of Applied Physics, Zaragoza University, 50009 Zaragoza, Spain; ^6^Department of Gastroenterology, Lozano Blesa University Hospital, C/San Juan Bosco 15, 50009 Zaragoza, Spain

## Abstract

*Purpose*. To evaluate the dose effect of vitamin K_3_ on wound healing mechanisms. *Methods*. Conjunctival fibroblasts were incubated for 24 hours. An artificial wound was made and the cells were incubated with fresh medium plus doses of vitamin K_3_ to be tested. Wound repair was monitored at 0, 18, 24, and 48 hours. Proliferation was measured in actively dividing cells by [^3^H]thymidine uptake. Six different groups were tested: group 1/no drugs added, group 2/ethanol 0.1%, group 3/vitamin K_3_ 1 mg/L, group 4/vitamin K_3_ 2 mg/L, group 5/vitamin K_3_ 4 mg/L, and group 6/vitamin K_3_ 6 mg/L. Each experiment was carried out in triplicate and 4 times. *Results*. There were no differences among groups at the initial time. *In vitro* wound repair was slower in groups 4, 5, and 6. There were no differences between control and ethanol groups and between control and vitamin K_3_ 1 mg/L groups. Fibroblast mitogenic activity was statistically decreased in all vitamin K groups; statistical differences were found among vitamin K_3_ 1 mg/mL and higher doses too. In groups 5 and 6, cellular toxicity was presented. *Conclusions*. Vitamin K_3_ is able to inhibit fibroblast proliferation. Vitamin K_3_ 2 mg/L or higher doses inhibit wound healing repair, exhibiting cellular toxicity at 4 and 6 mg/L.

## 1. Introduction

Antimetabolites and other fibroblast inhibitor drugs have been shown to enhance the success rate of filtering surgery although, depending on the dose, they can lead to severe complications and may result in the failure of the surgery.

Corticosteroids [1–6] antiproliferative agents (5-fluoro-uracil and other fluoropyrimidines, taxol, doxorubicin, mycophenolate mofetil…, alone or in combination or with different delivery systems) [3–12], systemic, periocular, intraocular steroidal, and nonsteroidal anti-inflammatory agents [5, 13–16], colchicine [8], daunomycin [8], tissue plasminogen activator [17], heparin [12, 18–20], interferon-gamma [21, 22], calcium channel blockers [23], prolyl and lysyl hydroxylase inhibitors [19, 24–26], retinoic acid [27, 28], alpha-tocopherol [29–31], disintegrins [32], siRNA-PKC*α* [33]… are some of the useful drugs that have been used in the treatment of conditions such as proliferative vitreoretinopathy, bleb scarring after trabeculectomy, and other disorders with cell proliferation (progressive conjunctival or extraocular cicatrization).

Vitamin K_3_ (menadione, 2-methyl-1,4-naphthoquinone) has been used as antihemorrhagic agent. Its ability to inhibit proliferation of tumor cells has already been reported; its activity has been demonstrated in human tumor stem cell and it is used in clinical trial for advanced malignancies acting in different pathways and has also been related to other oxidative stress processes at the eye level as cataract formation… [34–40]. Liu et al. reported that this drug could inhibit proliferation of rabbit conjunctive cells [41].

The aim of this study was to evaluate and to compare the antiproliferative properties of vitamin K_3_ in cultured human fibroblasts.

## 2. Methods

### 2.1. Material

All supplies for cell culture were purchased from Nunc (Roskilde, DK). Dulbecco's Modified Eagles Medium (DMEM), phosphate buffer saline (PBS), fetal calf serum (FCS), and antibiotics-antifungals were purchased from GIBCO (Madison, WI). [methyl-^3^H]thymidine was purchased from Amershm Iberica (Madrid, Spain). 2-Methyl 1,4-Naphthoquinone (Menadione) (98%) was obtained from Sigma (St. Louis, MO). The drug was initially dissolved in 90% ethanol. This alcohol solution was then diluted into BSS to yield a final ethanol concentration of 0.1%.

### 2.2. Cell Cultures

Conjunctival fibroblasts were obtained from explants of a healthy adult subject who underwent ophthalmic surgery for retinal detachment. All subjects gave informed consent to participate in the study, which was conducted in accordance with the tenets of the Declaration of Helsinki, and the experimental protocol was approved by the local Ethics Committee of the Aragon Health Science Institute. Cells were cultured in uncoated plastic flasks in DMEM supplemented with antibiotics and antifungals (100 IU/mL penicillin, 100 *μ*g/mL streptomycin, and 0.25 *μ*g/mL amphotericin B), and 20% fetal calf serum (FCS) in a humidified atmosphere at 37 degrees Celsius and 5% CO_2_. The culture medium was changed every 3 day and the experiments were performed with cells obtained between the 5th and 8th passages.

### 2.3. Wounding Assays

**Figure 1:**
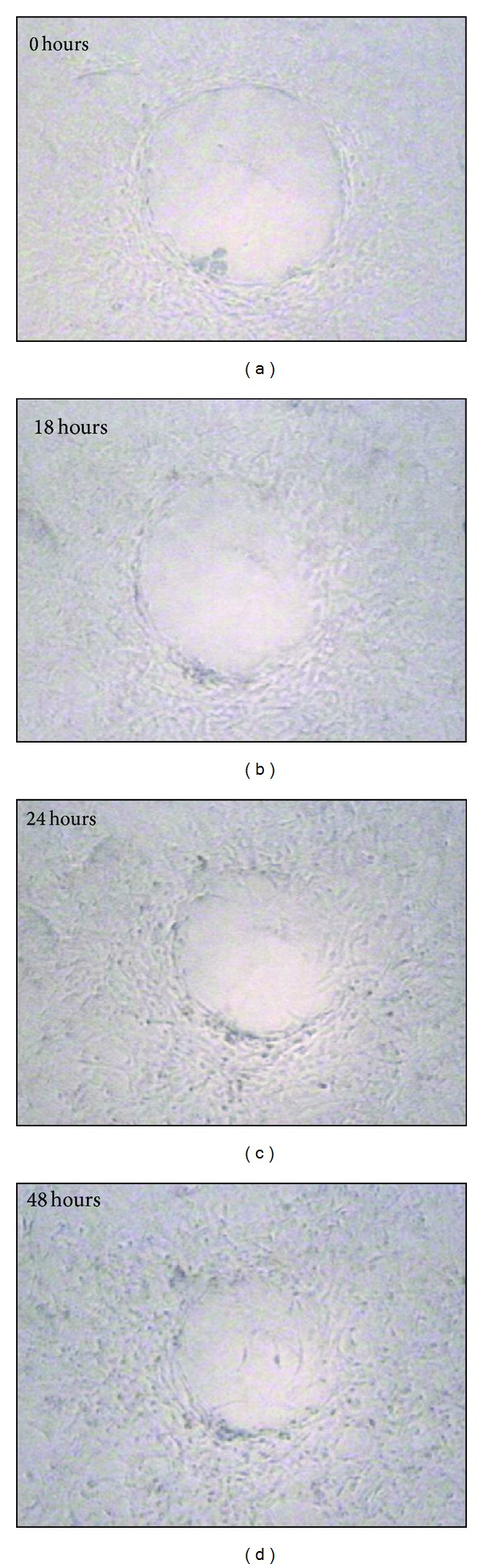
Phase-contrast microphotographs showing the process of wound healing in an ulcer treated with vitamin K_3_ 1 mg/L. Lesion time, 0 hour: *in vitro* ulcer after being produced in a confluent monolayer; 24 hours fibroblasts migrations was evident; 48 hours: some fibroblast were filling the ulcer area.

Wounding assays were performed using the method described by Sato and Watanabe [42]. An artificial wound was made by mechanical cell denudation with a rotating tip, as described in previous papers [26]. The wound repair process was monitored by two independent observers measuring the cell-free area (mm^2^) in a blind fashion, at different times: 0, 18, 24, and 48 hours (Figure 1). The cell-free area was quantified in the elliptic or circular shape wounds with homogeneous size. Then, the major and the minor axes were measured in a phase-contrast microscopy equipped with a calibrated visor. The area was calculated applying the mathematical formula: area = *A* × *B* × *π*/4, where *A* and *B* were the major and the minor axes, respectively.

### 2.4. Assay of Cell Mitogenic Activity

Freshly trypsinized fibroblasts were seeded in 24-well plates at a density of 15 × 10^4^ cells/well and were incubated for 24 hours in fresh medium and the drugs to be tested. Cells were labeled for the last 3 hours period with 1 *μ*Ci/mL of [methyl-^3^H]thymidine. After removing the media, the cells were washed 3 times with ice-cold PBS and then 2 times with 5% ice-cold trichloroacetic acid to precipitate the DNA. The precipitate was dissolved in 500 *μ*L 0.1 N NaOH and 0.1% sodium dodecyl sulfate. The extract was neutralized with 0.1 N HCl and radioactivity was counted in a liquid scintillation counter (1900 TR, Packard Instrument Company, Meriden, CT).

### 2.5. Groups of Treatment

There were 6 different treatment groups: group 1/control group: no drugs added, group 2/0.5 *μ*L of ethanol 10% (final ethanol concentration 0.1%); group 3/vitamin K_3_ 1 mg/L; group 4/vitamin K_3_ 2 mg/L; group 5/vitamin K_3_ 4 mg/L; group 6/vitamin K_3_ 6 mg/L.

### 2.6. Statistical Analysis

Each experiment was carried out in triplicate and at least 4 times. The results were expressed as mean ± standard deviation (*x*  ±  SD). Statistical significances between mean values were assessed with Mann-Whitney *U*-test. The probability level at which the Null Hypothesis was rejected was set at *P* < 0.05.

## 3. Results and Discussion

Wound healing in some ocular diseases and surgeries, as mucous-cutaneous diseases or after glaucoma filtering surgery, or complicated retinal detachment, is one of the problems that needs to be solved. We were studying the effect of vitamin K on wound healing and its possible toxicity.

The wound area is presented in Table 1. There were no differences among the groups at the initial time (Table 1). The mean size was 0.586 mm^2^ ± 0.082. There were no differences between control group and ethanol group. Ethanol 0.1 mg/mL did not show effect on fibroblast migration and proliferation. No toxic effect had been related to its use. Vitamin K is a liposoluble drug. This fact can be remarkable in order to use it to assist the effect of the silicone oil on complicated retinal detachments.

Wound healing process can be divided into three phases: inflammation, proliferation, and modulation of the scar. This process begins immediately after the injury. The fibroblast proliferation appears after 24 hours [43]. In this experimental model of wound healing, we evaluate the fibroblast migration 18 hours after the ulcer has been done. In the 24 hours' time migration and proliferation are evident.

Vitamin K_3_ at the doses 2, 4, and 6 mg/L significantly decreased the speed of wound repair during the experiment. There were no differences between control group and vitamin K_3_ 1 mg/L (Table 2). Vitamin K_3_ at the doses 2, 4, and 6 mg/L inhibited the cell migration and proliferation and showed slower closure of the wounds than the other groups (from 18 hours on). Liu et al. found that most of the cells died at concentrations of 7.5 mg/L; the concentration of 4.0 mg/L inhibited fifty percent of the cellular growth. The cellular border became clearer and some cells started to die at 5 mg/L [41].

Fibroblast mitogenic activity was significantly inhibited by all vitamin K_3_ doses. There were differences between vitamin K_3_ 1 mg/mL and all the others vitamin K_3_ groups. In this study, vitamin K_3_ at 1 mg/L did not show differences with the control group in the speed of wound repair. Fibroblast mitogenic activity was inhibited by all doses of vitamin K_3_; differences were found among vitamin K_3_ 1 mg/mL and the greater doses. Vitamin K_3_ 1 mg/mL is able to inhibit fibroblast mitogenic activity with no influence in wound repair; this effect has probably been counteracted by its no migration inhibitory effect.

In our study, doses of 4 mg/L induced great cellular alterations. Vitamin K_3_ 4 mg/L and 6 mg/L induced cellular toxicity. Cells presented changes in their morphology, which characterized apoptosis, including nuclear and cytoplasmic condensation with intact plasma membrane cell. They lost their adherence to the plate, showing a growing ulcer throughout the time being the ulcer sizes larger than in the other groups. MMC and 5-FU are also able to induce apoptosis in cultured tenon's fibroblast [44].

The application of experimental data derived from cell cultures to clinical use has limitations. Variables such as bioavailability, diffusional barriers, metabolic inactivation, excretion, drug resistance, and enzyme induction prohibit simple extrapolation of cell culture data to human diseases. Nevertheless, this basic approach to drug selection is invaluable.

The mechanism of cytotoxicity of vitamin K_3_ is not well known and it has been the focus of multiple papers. The drug is able to affect the cell by two mechanisms. One is its ability to disturb the intracellular calcium flux and calcium-dependent potassium flux [45]. The other is that its chemical transformation within the cell may generate reactive oxygen species and potentially deplete intracellular glutathione [46]. Effects on different cells have been described such as inhibition of PTP-1B in keratinocytes [47], induction of tumor cell death through hydrogen peroxide generation, and regulation of the expression of G1 phase-related cell cycle molecules [48, 49].

We can conclude that all the studied doses of vitamin K were able to inhibit fibroblast mitogenic activity. Vitamin K_3_, at 2 mg/L or higher doses, interfere the mechanisms of cell repair, delaying the wound healing process in this *in vitro* model. Vitamin K_3_ at 4 and 6 mg/L in cell culture showed fibroblast toxicity. The drug could be considered an alternative to the drug treatment and prevention of exaggerated scarring in some ocular diseases.

## Figures and Tables

**Table 1:** Measurement of the wound areas (values are expressed as mean ± SD).

AREA (mm^2^)	0 hours	18 hours	24 hours	48 hours
Control	0.599± 0.118	0.332± 0.108	0.267± 0.118	0.069± 0.066
K_3_ 1 mg/L	0.569± 0.099	0.405± 0.056	0.356± 0.048	0.125± 0.073
K_3_ 2 mg/L	0.669± 0.120	0.672± 0.095	0.619± 0.082	0.459± 0.232
K_3_ 4 mg/L	0.554± 0.162	0.771± 0.096	>1 mm^2^	>1 mm^2^
K_3_ 6 mg/L	0.582± 0.110	0.774 ± 0.039	>1 mm^2^	>1 mm^2^

**Table:** (a)

0 hours	Control	K_3_ 1 mg/L	K_3_ 2 mg/L	K_3_ 4 mg/L	K_3_ 6 mg/L
Control		NS	NS	NS	NS
K_3_ 1 mg/L			NS	NS	NS
K_3_ 2 mg/L				NS	NS
K_3_ 4 mg/L					NS

**Table:** (b)

18 hours	Control	K_3_ 1 mg/L	K_3_ 2 mg/L	K_3_ 4 mg/L	K_3_ 6 mg/L
Control		NS	S	S	S
K_3_ 1 mg/L			S	S	S
K_3_ 2 mg/L				NS	NS
K_3_ 4 mg/L					NS

**Table:** (c)

24 hours	Control	K_3_ 1 mg/L	K_3_ 2 mg/L	K_3_ 4 mg/L	K_3_ 6 mg/L
Control		NS	S	S	S
K_3_ 1 mg/L			S	S	S
K_3_ 2 mg/L				S	S
K_3_ 4 mg/L					NS

**Table:** (d)

48 hours	Control	K_3_ 1 mg/L	K_3_ 2 mg/L	K_3_ 4 mg/L	K_3_ 6 mg/L
Control		NS	S	S	S
K_3_ 1 mg/L			S	S	S
K_3_ 2 mg/L				S	S
K_3_ 4 mg/L					NS
